# A Transgenic *Drosophila* Model Demonstrates That the *Helicobacter pylori* CagA Protein Functions as a Eukaryotic Gab Adaptor

**DOI:** 10.1371/journal.ppat.1000064

**Published:** 2008-05-16

**Authors:** Crystal M. Botham, Anica M. Wandler, Karen Guillemin

**Affiliations:** Institute of Molecular Biology, University of Oregon, Eugene, Oregon, United States of America; Stanford University, United States of America

## Abstract

Infection with the human gastric pathogen *Helicobacter pylori* is associated with a spectrum of diseases including gastritis, peptic ulcers, gastric adenocarcinoma, and gastric mucosa–associated lymphoid tissue lymphoma. The cytotoxin-associated gene A (CagA) protein of *H. pylori*, which is translocated into host cells via a type IV secretion system, is a major risk factor for disease development. Experiments in gastric tissue culture cells have shown that once translocated, CagA activates the phosphatase SHP-2, which is a component of receptor tyrosine kinase (RTK) pathways whose over-activation is associated with cancer formation. Based on CagA's ability to activate SHP-2, it has been proposed that CagA functions as a prokaryotic mimic of the eukaryotic Grb2-associated binder (Gab) adaptor protein, which normally activates SHP-2. We have developed a transgenic *Drosophila* model to test this hypothesis by investigating whether CagA can function in a well-characterized Gab-dependent process: the specification of photoreceptors cells in the *Drosophila* eye. We demonstrate that CagA expression is sufficient to rescue photoreceptor development in the absence of the *Drosophila* Gab homologue, Daughter of Sevenless (DOS). Furthermore, CagA's ability to promote photoreceptor development requires the SHP-2 phosphatase Corkscrew (CSW). These results provide the first demonstration that CagA functions as a Gab protein within the tissue of an organism and provide insight into CagA's oncogenic potential. Since many translocated bacterial proteins target highly conserved eukaryotic cellular processes, such as the RTK signaling pathway, the transgenic *Drosophila* model should be of general use for testing the *in vivo* function of bacterial effector proteins and for identifying the host genes through which they function.

## Author Summary

Like many pathogens, the human gastric bacterium *Helicobacter pylori* orchestrates infection through the activity of proteins that it translocates into host cells. The *H. pylori* translocated protein, CagA, which shares no homology to any other proteins, is a significant risk factor for *H. pylori*–associated diseases including gastric cancer. Experiments in tissue culture cells have shown that CagA can activate SHP-2 phosphatase, a component of the receptor tyrosine kinase signaling pathway. Based on this activity, CagA has been proposed to function as a mimic of Gab proteins that serve as adaptors in this signaling pathway. We have developed a transgenic *Drosophila* model to test this hypothesis in the tissues of an organism. We demonstrate that CagA can substitute for Gab and restore developmental defects caused by the loss of the *Drosophila* Gab, including promoting photoreceptor specification in the developing eye. Furthermore, we show that CagA functions similarly to Gab because it requires the *Drosophila* SHP-2 to exert its effect on photoreceptor development. Our transgenic *Drosophila* model provides new insight into CagA's activity in tissues and will allow us to identify host factors through which CagA functions to manipulate cellular signaling pathways and promote disease.

## Introduction

The human pathogen, *Helicobacter pylori*, infects the stomachs of at least half the world's population and chronic infection is associated with the development of diseases such as gastritis, peptic ulcers and gastric cancer [1]. A major virulence determinant of *H. pylori* is the cytotoxin associated gene A (CagA) which is translocated into host cells via a type four secretion system (reviewed in [2]). Inside host cells, CagA is phosphorylated by Src family kinases on tyrosines contained in repeated five-amino acid motifs (EPIYA) in CagA's carboxyl terminus. Phosphorylated CagA disrupts receptor tyrosine kinase (RTK) signaling pathways by directly activating Src homology 2 (SH2) domain containing tyrosine phosphatase (SHP-2) (reviewed in [3]). Normally SHP-2 is activated by the scaffolding adaptor Grb2-associated binder (Gab) proteins, thereby amplifying RTK signaling pathways to control cell growth, differentiation and survival (reviewed in [4]). The Gab proteins occupy a pivotal position in RTK signaling pathways by interacting directly with RTKs such as the c-Met receptor of the Hepatocyte growth factor/Scatter factor (HGF/SF) as well as downstream cytoplasmic proteins including SHP-2, v-crk sarcoma virus CT10 oncogene homolog (avian)-like (Crk(L)), and Growth factor receptor-bound protein 2 (Grb2) (reviewed in [5],[6],[7]). Although CagA shares no sequence similarity with Gab proteins, CagA has been shown to activate SHP-2 in tissue culture cells, resulting in cell elongation [8],[9]. Similarly, in tissue culture cells CagA has been found to associate with c-Met, Crk(L) and Grb2 [10],[11],[12]. Based on these interactions, CagA has been hypothesized to mimic Gab proteins and to function as an oncogene by over-activating RTK signaling [13]. The significance of CagA's interactions with RTK signaling pathway proteins, however, has only been explored in tissue culture cells.

We have developed transgenic *Drosophila* with inducible CagA expression as a model to understand CagA's mechanisms of action in complex epithelial tissues. In order to test the hypothesis that CagA can function as a Gab substitute, we investigated CagA activity in a well-characterized Gab-dependent process, the specification of photoreceptors in the *Drosophila* eye [14],[15],[16]. The *Drosophila* compound eye, whose crystalline array of facets or ommatidia are exquisitely sensitive to perturbations in cell specification, has been used as a powerful system for the discovery and genetic analysis of RTK signaling components [17],[18]. *Drosophila* RTK signaling proteins are highly conserved with their mammalian orthologues and oncogenic mutations in these proteins, such as those that constitutively activate RTK receptors or their downstream effectors, function similarly in both *Drosophila* and mammalian cells [19]. The *Drosophila* model also offers elegant tools for genetic manipulations including the UAS/GAL4 system [20] for expression of transgenes in a tissue specific manner, the FLP/FRT system for the generation of somatic mutant clones [21], and null mutations in most RTK signaling pathway members, which allow us to probe the *in vivo* requirements for CagA's activation of RTK signaling pathways. Finally, *Drosophila* are amenable to forward genetic approaches that will facilitate the discovery of host factors required for CagA function in eukaryotic cells [22].

RTK signaling is required for multiple steps of *Drosophila* photoreceptor development. The *Drosophila* epidermal growth factor receptor (EGFR) is necessary for cell proliferation in the early eye imaginal disc, cell survival in the differentiating region of the disc behind the morphogenetic furrow, and recruitment of all photoreceptors except R8 [23]. A second RTK, Sevenless (SEV) is required exclusively for the R7 photoreceptor to adopt the appropriate fate, as opposed to becoming a nonneuronal cone cell [24] (reviewed in [25]). The *Drosophila* Gab adaptor, Daughter of Sevenless (DOS) is required for full signaling through both the EGFR and SEV pathways [16]. Clones of eye imaginal cells lacking DOS activity fail to proliferate and produce few photoreceptors, similar to clones lacking EGFR [16],[26],[27]. The EGFR pathway is required additionally for multiple aspects of *Drosophila* development [28].

Here we show that CagA can substitute for the *Drosophila* Gab adaptor, DOS, and rescue phenotypes associated with loss of *dos*, including larval lethality and photoreceptor differentiation. We further demonstrate that CagA functions through the *Drosophila* SHP-2 homologue, Corkscrew (CSW) similar to Gab. Our work demonstrates the power of using a genetically tractable system like *Drosophila* to dissect the mechanism of action of a prokaryotic protein that modulates a conserved eukaryotic signaling pathway.

## Results

### CagA is phosphorylated, cortically-localized in *Drosophila* cells and disrupts eye development

To determine if the *Drosophila* system would be useful for dissecting the molecular mechanism of CagA-induced activation of RTK signaling, we examined whether CagA exhibited similar properties when expressed in *Drosophila* tissue to those previously observed in mammalian tissue culture cells. We used P-element mediated transgenesis to generate *Drosophila* with a transgene encoding an N-terminal hemagglutinin (HA) tagged CagA under control of the yeast GAL4 upstream activating sequence (*UAS-CagA*). Additionally, we generated transgenic flies with a mutated version of CagA lacking the EPIYA tyrosine phosphorylation motifs (*UAS-CagA^EPISA^*). These transgenic flies were crossed to flies that expressed the GAL4 transcription factor under tissue-specific or inducible promoters to express CagA in specific cells and at specific times during development. In the experiments described here, the *GMR-GAL4* line was used to express CagA in all cells of the developing imaginal eye disc after the morphogenetic furrow.

**Figure 1:**
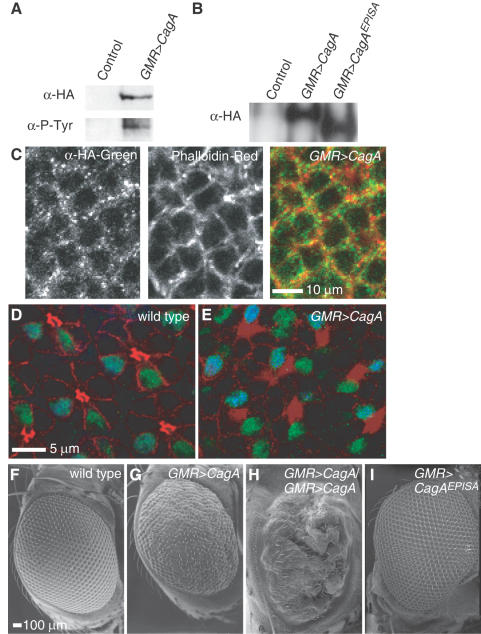
CagA is phosphorylated, associates with the cortex in *Drosophila* cells and disrupts eye development. (A) *UAS-CagA* was expressed in the *Drosophila* eye with *GMR-GAL4*. CagA protein (α-HA) was tyrosine phosphorylated (α-P-Tyr). Controls expressed only *GMR-GAL4*. (B) The CagA and CagA^EPISA^ proteins were expressed in the eye to similar levels. (C) CagA (α-HA, green) localized to the cortex (phalloidin, red) of cells in the larval eye disc. Wild type (D) and *UAS-CagA/GMR-GAL4* (E) pupal retinas were stained with MAb 24B10 (red) to outline the photoreceptors, and antibodies to visualize the R7 (green) and R8 (cyan) photoreceptors. Scanning electron microscope micrographs of adult eyes from flies with (F) one copy of *GMR-GAL4* and no *UAS* transgene, (G) one copy of *UAS-CagA*, (H) two copies of *UAS-CagA* and (I) one copy of *UAS-CagA^EPISA^*.

Western analysis of anti-HA affinity purified proteins from heads of adult *UAS-CagA/GMR-GAL4* flies showed that CagA was expressed (α-HA) and phosphorylated (α-P-Tyr, Figure 1A). Similar to CagA's distribution in tissue culture cells [8],[29], we showed in the *Drosophila* eye disc CagA was localized predominantly to the cell cortex (Figure 1C). Examination of the cellular morphology of the pupal retina revealed that CagA expression caused disorganization of the epithelium. The wild type retinal epithelium is organized into regular cell clusters, each containing a single R7 and R8 photoreceptor (Figure 1D). In retina expressing CagA, the normal cell shapes and neighbor relationships were perturbed (Figure 1E), similar to CagA-dependent epithelial disorganization observed in mammalian tissue culture monolayers [29],[30]. When we examined the eyes of adult flies expressing a single copy of CagA with *GMR-GAL4*, we observed a perturbation of the normal crystalline array of the ommatidia (compare wild type, Figure 1F, with CagA expression, Figure 1G). Expression of two copies of the *UAS-CagA* transgene dramatically enhanced the eye phenotype, indicating that the developmental pathways disrupted were sensitive to the amount of CagA expressed (Figure 1H). Expressing one copy of the CagA mutant lacking the tyrosine phosphorylation sites (CagA^EPISA^) did not perturb the crystalline array of the adult eye to the extent caused by wild type CagA (Figure 1I) even though the CagA^EPISA^ protein was expressed at similar levels as CagA (Figure 1B). Dose dependent perturbations of *Drosophila* eye patterning, as observed with CagA expression, have been used as the basis for genetic screens for modifiers of the rough eye phenotype to elucidate several signaling pathways, including RTK pathways. [17],[31]

### CagA can substitute for the *Drosophila* Gab

**Figure 2:**
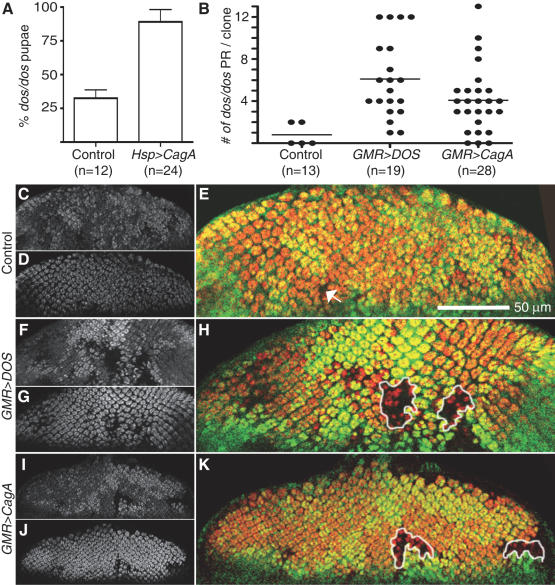
CagA can substitute for the *Drosophila* Gab. (A) Few homozygous *Hsp-GAL4/+; dos/dos* individuals survived to the pupal stage (control) measured as *dos/dos* pupae that developed as a percentage (%) of expected pupae that should develop if *dos* mutants were fully viable. Significantly more *UAS-CagA/Hsp-GAL4; dos/dos* pupae were recovered (Chi squared, p value<0.02). n = number experiments completed, with between 150-450 pupae examined in each experiment. Error bars indicate standard error. The *dos/dos* mitotic clones (marked by the absence of GFP (C, F, I, and green staining in E, H, K)), were induced using the FLP/FRT system in different genetic backgrounds, and photoreceptors were visualized with ELAV staining (D, G, J, and red staining in E, H, K). (B) The number of photoreceptors (PR) per *dos/dos* clones is shown for a representative experiment (n = number of eye discs examined). An area lacking GFP was counted as a clone if it was large enough to normally contain at least one ommatidium. (C–E) In control flies expressing *GMR-GAL4*, few *dos/dos* clones were observed. Any clones were miniscule (arrow in E) and contained few photoreceptors. Photoreceptors development was rescued by expression of either *GMR-GAL4*; *UAS-DOS* (F–H) or *GMR-GAL4; UAS-CagA* (I–K) as indicated by the formation of larger *dos/dos* clones containing several photoreceptors (clones are outlined in H and K).

To test the hypothesis that CagA functions as a prokaryotic mimic of eukaryotic Gab proteins, we asked whether CagA expression could rescue phenotypes caused by the loss of the *Drosophila* Gab, DOS. DOS functions downstream of multiple RTKs during development, and homozygous *dos* loss-of-function mutants rarely develop into pupae and never survive to adulthood [16]. Rescue of *dos* mutants' lethality has been used as an *in vivo* assay to determine the function of specific domains of DOS [26]. We therefore determined the percentage of *dos* homozygous mutants that survived to the pupal stage of development with or without CagA expressed ubiquitously with temporal precision using the heat shock inducible *Hsp-GAL4*. The frequency of *dos* homozygous mutants was scored as a percentage of expected pupae that should develop if the *dos* mutants showed no lethality defect. As expected, a low percentage (33%) of homozygous *dos* mutant pupae expressing only *Hsp-GAL4* were observed (Figure 2A). When CagA was expressed, we observed a significant increase to 89% of the pupae developing that lacked *dos* (Figure 2A). These results indicate that CagA can substitute for essential functions of DOS during *Drosophila* development.

To specifically test whether CagA could substitute for Gab in photoreceptor development, we generated mitotic *dos/dos* clones within the eye using the FLP/FRT recombinase system [27],[32]. In these experiments the *dos* mutation was recombined onto a chromosome arm containing a centromere proximal FRT recombination site and maintained in trans to a chromosome containing the same FRT site as well as a *GFP* transgene. By expressing FLP recombinase in the developing eye we induced mitotic recombination between FRT sites, which generated clones of homozygous cells (*+/+* and *dos*/*dos*) in an otherwise heterozygous background (*dos/+*). The *dos/dos* mutant cells were distinguished by their lack of GFP, and the photoreceptors were visualized by staining for the photoreceptor-specific protein ELAV. As previously reported [16],[26] the *dos/dos* clones rarely contained photoreceptors and were composed of very few cells (Figure 2B–E), due to the dual requirements for EGFR signaling in cell survival and photoreceptor specification [23]. As expected, expression of DOS with *GMR-GAL4* in *dos/dos* cells resulted in much larger clones with increased numbers of photoreceptors (Figure 2B, F–H). Expression of CagA in *dos/dos* cells was able to rescue clone size and photoreceptor development similarly to expression of DOS with the same driver (Figure 2B, I–K). Two independent *dos* mutants gave similar results (Figure 2 and data not shown). These data demonstrate that CagA can substitute for DOS during the development of photoreceptors.

### CagA's specification of photoreceptors requires SHP-2/CSW

**Figure 3:**
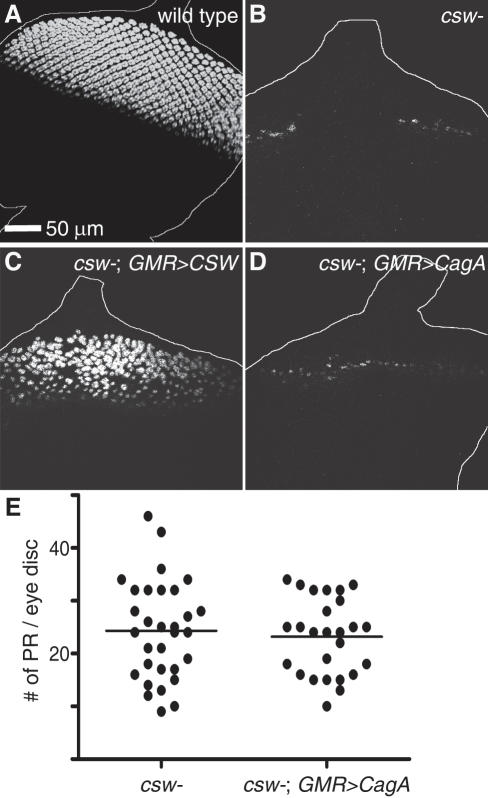
CagA's specification of photoreceptors requires SHP-2/CSW. (A) Wild type larval eye discs contain thousands of photoreceptors, which were visualized by anti-ELAV staining. (B) Few photoreceptors develop in *csw* mutant larval eye discs. (C) Expression of *UAS-CSW* using *GMR-GAL4* in the *csw* mutant partially rescued the lack of photoreceptor development but expression of *UAS-CagA* did not (D). (E) There was no significant difference (T-test, p value>0.2) in the number of photoreceptors (PR) that developed in *csw* mutants with or without CagA expression.

We predicted that if CagA functions similarly to Gab, then CagA would require the downstream signaling molecule SHP-2/CSW to promote photoreceptor development. As a downstream component of RTK pathways, CSW is required for photoreceptor development [17]. In contrast to wild type larval eye discs, in which thousands of photoreceptors are specified (Figure 3A), in larval eye discs of *csw* null mutants only a few photoreceptors develop along the morphogenetic furrow (Figure 3B, E) as described previously [33]. The residual photoreceptors in the *csw* eye discs were mostly R8 cells (data not shown), the only photoreceptor class that does not require RTK signaling for its specification [23]. A significant increase in photoreceptor number could be achieved in the *csw* mutant eye discs by expression of *UAS-CSW* with *GMR-GAL4* (Figure 3C) or *Hsp-GAL4* (data not shown). However, expression of CagA from multiple different transgenic lines using either *GMR-GAL4* or *Hsp-GAL4* failed to increase the number of photoreceptors in two different *csw* null mutants (Figure 3D, E, data not shown). These results argue that CagA, like DOS, requires SHP-2/CSW to promote photoreceptor development.

## Discussion

We used a transgenic *Drosophila* system to test the hypothesis that *H. pylori's* virulence factor CagA can substitute for the Gab adaptor in RTK signaling pathways. This system is ideal for these studies because RTK signaling pathway components can be genetically manipulated, resulting in interpretable phenotypic consequences for tissue development. First, we have demonstrated that CagA in *Drosophila* tissue is phosphorylated, that it associates with the cell cortex, and that its expression causes epithelial disorganization as in mammalian tissue culture cells. Second, we have provided genetic evidence that CagA can substitute for Gab by demonstrating that CagA expression restores larval viability and photoreceptor development in mutants lacking the *Drosophila* Gab, DOS. Our inability to rescue *dos* mutants to adulthood with CagA expression may be due to differences in RTK activation or to non-overlapping functions of Gab and CagA. Indeed too much CagA expression (using an *actin-GAL4* driver) is lethal to flies (unpublished results), which is not the case for ubiquitous expression of DOS [26]. Third, our genetic epistasis analysis with mutants lacking *csw* has shown that CagA functions through the *Drosophila* SHP-2 homologue, similar to results from tissue culture experiments [8],[9].

RTK signaling is essential for several fundamental biological processes and erroneous signaling can promote tumor formation [19]. Gain-of-function mutations of SHP-2 have been established as oncogenic in numerous leukemia types as well as other diseases like Noonan's Syndrome [4],[34],[35]. Over-expression of the Gab scaffolding adaptor proteins is associated with the development of several types of cancers, including breast cancer [6],[7] and gastric cancer [36]. The specific cancers that develop as a result of these mutations reflect tissue sensitivities to increased Gab and SHP-2. In the case of *H. pylori* infection, CagA provides a tissue specific activation of RTK signaling that can precipitate events leading to gastric carcinogenesis [37], as suggested by a recent report of CagA-expressing transgenic mice [38].

Our approach of examining the cellular effects of CagA expression in *Drosophila* tissue takes advantage of the fact that bacterial proteins frequently target essential, highly conserved cell-signaling pathways. *Drosophila* has been employed traditionally as a model organism for dissecting signaling pathways in development, but in recent years it has also proven useful in understanding host-pathogen interactions (reviewed in [39],[40]), and in one instance has been used as a heterologous system for expression of the bacterial toxins, anthrax lethal and edema factors [41]. Here we have exploited *Drosophila* eye development to demonstrate CagA's capacity to function as a RTK adaptor. Future studies using this transgenic *Drosophila* model will allow us to better understand the cellular and tissue-wide consequences of CagA's disruption of eukaryotic signaling pathways and to identify candidate host factors through which CagA functions.

## Materials and Methods

### Construction of *UAS-CagA* and *UAS-CagA^EPISA^*

CagA cDNA was amplified from genomic DNA from *H. pylori* G27. The CagA^EPISA^ (lacking EPIYA tyrosine phosphorylation motifs) cDNA was amplified from a plasmid provided by Manuel Amieva (originally from Markus Stein [42]). CagA^EPISA^ lacks the tyrosines in the four 5-amino acid motifs, EPIYA, which are phosphorylated by host kinases (point mutations at nucleotide 2684 [A→C] and 2740 [A→C] and a deletion at nucleotide 2878 to 3082). CagA and CagA^EPISA^ were cloned into a modified pUAST vector with an N-terminal hemagglutinin (HA) tag (provided by Chris Q. Doe). Transgenic lines were generated by injecting Qiagen-purified plasmid DNA into *y,w^1118^* embryos. Several independent transformant lines were established for each construct.

### *Drosophila* Strains

Genetic null alleles of *csw* (*csw^C114^* and *csw^13-87^*) and *dos* (*dos^1.46^* and *dos^2.46^*) were obtained from Michael Simon. The *UAS-DOS* strain was from Thomas Raabe and the *UAS-CSW* strain (*UAS-flgcsw[WTCIM]*) from Lizabeth Perkins. *UAS-CagA* and *UAS-CagA^EPISA^* (lacking EPIYA tyrosine phosphorylation motifs) transgenes were expressed in the eye using *P{w[+mC] = GAL4-ninaE.GMR}12* (*GMR-GAL4*, Bloomington Stock Center (BSC) # 1104). *P{GAL4-Hsp70.PB}2* (*Hsp-GAL4*, BSC # 2077) was used for heat-shock inducible expression of transgenes.

### Scanning Electron Microscopy

Fly heads were fixed overnight at 4°C in 2% gluteraldehyde in 0.1 M sodium cacodylate buffer (pH 7.2) and dehydrated through an ethanol series (30%, 50%, 70%, 80%, 90% 95%, three times in absolute ethanol) at room temperature for 10 minutes in each solution. Samples were critically point dried, sputter coated with gold and viewed using a JEOL 6400 SEM.

### Larval Eye Discs

Eye imaginal discs were dissected from third instar wandering larvae, fixed for 30 minutes (4% formaldehyde, 0.1 M PIPES (pH 6.9), 0.3% Triton X-100, 2 mM EGTA, 1 mM MgSO_4_). Discs were washed (0.3% Triton X-100 in phosphate buffered saline, PBS) and blocked for one hour (1% BSA, 0.3% Triton X-100 in PBS). Primary antibodies included rat anti-ELAV 1∶10 (05HB 7E8A10, from Chris Q. Doe), rat anti-HA 1∶100 (Roche) and chicken anti-GFP 1∶2,000 (Chemicon). Secondary antibodies included anti- rat conjugated Rhodamine Red 1∶200 (Jackson ImmunoResearch), anti-rat conjugated AlexaFluor 488 1∶200 (Molecular Probes), anti-mouse conjugated Cy3 1∶200 (Jackson ImmunoResearch) and anti-chicken conjugated Cy2 1∶100 (Jackson ImmunoResearch). Phalloidin conjugated to Tetramethyl Rhodamine Iso-Thiocyanate (TRITC, Sigma Aldrich, 1∶500) was used to stain F-actin. Imaginal discs were visualized using a Nikon TE2000 U with C1 Digital Eclipse confocal microscope.

### Pupal Retinas

Wandering third instar larvae were placed at 25°C and approximately 50 hours later the pupal retinas were dissected (50% pupal stage). Retinas were dissected in PBS, fixed for 20 minutes (4% paraformaldehyde in PBS) and washed three times in PBT (0.5% Triton X-100 in PBS). Retinas were blocked at least 15 minutes in 10% normal goat serum in PBT. Antibodies were diluted in the blocking solution. Primary antibodies included mouse MAb 24B10 which stains all photoreceptors and their axons [43] (Developmental Studies Hybridoma Bank, 1∶200), rabbit anti-SAL, which stains R7 and R8 nuclei (also called SPALT, provide by Reinhard Schuh [44], 1∶100), guinea pig anti-SENSELESS, which stains R8 nuclei (proved by Hugo Bellen [45], 1∶1000). Secondary antibodies from Molecular Probes included AlexaFluor 555 conjugated anti-mouse, AlexaFluor 488 conjugated anti-rabbit and AlexaFluor 633 conjugated anti-guinea pig, which were all used at 1∶250. Pupal retinas were visualized using a Leica TCS SP5 confocal microscope.

### Western Analysis

Fly heads were collected by flash freezing adult flies in liquid nitrogen, shaking the flies in a conical tube, and then separating the heads from the bodies using a mesh sieve. Heads (∼1.5 mL) were homogenized in ice cold lysis buffer (50 mM Hepes, 150 mM NaCl, 1 mM EDTA, 1 mM Na_3_VO_4_, 0.5% Triton X-100 and Complete protease inhibitors [Roche]) and then centrifuged at 16,000 G for 5 minutes. Supernatant from the lysate solution (1.5 mL) was added to 50 µL anti-HA Affinity Matrix (Roche) which was incubated overnight at 4°C with gentle agitation. The anti-HA affinity matrix was washed 4 times with ice-cold lysis buffer. CagA was eluted from the matrix by boiling in 100 uL sample loading buffer and separated using manufactures protocols for 7% NuPAGE® Novex Tris-Acetate gels, transferred to polyvinylidene difluoride membranes, blocked overnight at 4°C (200 mM Tris pH 7.5, 100 mM NaCl, 0.1% Tween-20 and 3% BSA (Fisher)), probed using appropriate antibodies and detected using enhanced chemiluminescene (ECL plus, Amersham Biosciences). Mouse anti-HA was used at 1∶1,000 (Babco). Mouse anti-phospho tyrosine was used at 1∶2,000 (Cell Signal Technologies). Horseradish peroxidase-conjugated sheep anti-mouse (Amersham Biosciences) was used at 1∶5,000.

### *dos* Assays

*Hsp70-GAL4* balanced over *CyO, P{Ubi-GFP}* with *dos^2.42^* over *TM3, P{Act-GFP}, Ser* were crossed to *dos^1.46^/TM3, P{Act-GFP}, Ser* (negative control) or *UAS-CagA; dos^1.46^/TM3,P{Act-GFP}, Ser*. Progeny were raised at 30°C and pupae were examined for GFP florescence using a Stemi SV 11 Apo Zeiss microscope. The number of non-GFP expressing progeny was scored as a percentage of the total number of pupae that developed per bottle and averaged across bottles of the same genotype. At least 12 bottles were scored per cross with between 150–450 pupae examined per bottle.

The FLP/FRT recombinase system was used to induce somatic clones in the eye [27]. Males *y w,ey-FLP 3.5/Y; GMR-GAL4; FRT2, dos^1.46^/CyO-TM6B* were crossed to *P{ey-FLP.N}6, ry506 (BSC #5577); P{Ubi-GFP.nls}3L1 P{Ubi-GFP.nls}3L2 P{FRT(whs)}2A (BSC #5825)* (negative control) or *UAS-DOS; P{Ubi-GFP.nls}3L1 P{Ubi-GFP.nls}3L2 P{FRT(whs)}2A* (positive control). Male *GMR-GAL4, UAS-CagA; FRT2, dos^1.46^/CyO-TM6B* were crossed to *P{ey-FLP.N}6, ry506; P{Ubi-GFP.nls}3L1 P{Ubi-GFP.nls}3L2 P{FRT(whs)}2A*. Imaginal eye discs were stained with anti-ELAV and anti-GFP antibodies.

### *csw* Assay

Two genetic null alleles of *csw* were used to examine if CagA could rescue loss of *csw*. The *csw^C114^* or *csw^13-87^* alleles were balanced over *FM7, P{Act-GFP}* with *GMR-GAL4* balanced over *CyO, P{Ubi-GFP}* on the second chromosome. These females were then crossed to males *y^1^w^1118^, P{Ubi-GFP.nls}X1 P{FRT(w^hs^)}9-2 (BSC # 5832)/Y* (negative control), *y^1^w^1118^, P{Ubi-GFP.nls}X1 P{FRT(w^hs^)}9-2/Y; UAS-CSW* (positive control) or *y^1^w^1118^, P{Ubi-GFP.nls}X1 P{FRT(w^hs^)}9-2/Y; UAS-CagA*. Eye imaginal discs were dissected from male larvae.
